# Perception of body shape and size without touch or proprioception: evidence from individuals with congenital and acquired neuropathy

**DOI:** 10.1007/s00221-021-06037-4

**Published:** 2021-02-12

**Authors:** R. Christopher Miall, Daria Afanasyeva, Jonathan D. Cole, Peggy Mason

**Affiliations:** 1 School of Psychology, University of Birmingham, Birmingham, B15 2TT UK; 2 Centre of Postgraduate Research and Education, Bournemouth University, Bournemouth, UK; 3 Department of Neurobiology, The University of Chicago, Chicago, IL USA

**Keywords:** Body representation, Somatosensation, Deafferentation, Sensory neuropathy

## Abstract

The degree to which mental representations of the body can be established and maintained without somatosensory input remains unclear. We contrast two “deafferented” adults, one who acquired large fibre sensory loss as an adult (IW) and another who was born without somatosensation (KS). We compared their responses to those of matched controls in three perceptual tasks: first accuracy of their mental image of their hands (assessed by testing recognition of correct hand length/width ratio in distorted photographs and by locating landmarks on the unseen hand); then accuracy of arm length judgements (assessed by judgement of reaching distance), and finally, we tested for an attentional bias towards peri-personal space (assessed by reaction times to visual target presentation). We hypothesised that IW would demonstrate responses consistent with him accessing conscious knowledge, whereas KS might show evidence of responses dependent on non-conscious mechanisms. In the first two experiments, both participants were able to give consistent responses about hand shape and arm length, but IW displayed a better awareness of hand shape than KS (and controls). KS demonstrated poorer spatial accuracy in reporting hand landmarks than both IW and controls, and appears to have less awareness of her hands. Reach distance was overestimated by both IW and KS, as it was for controls; the precision of their judgements was slightly lower than that of the controls. In the attentional task, IW showed no reaction time differences across conditions in the visual detection task, unlike controls, suggesting that he has no peri-personal bias of attention. In contrast, KS did show target location-dependent modulation of reaction times, when her hands were visible. We suggest that both IW and KS can access a conscious body image, although its accuracy may reflect their different experience of hand action. Acquired sensory loss has deprived IW of any subconscious body awareness, but the congenital absence of somatosensation may have led to its partial replacement by a form of visual proprioception in KS.

## Introduction

In this paper, we contrast two very rare yet related neurological conditions: the acquired loss of proprioception and touch in adulthood and the complete congenital absence of these senses. We compare individuals with these two conditions to each other and to normal controls to illuminate how representations of the body can be established and maintained without somatosensory input.

Normally, an internal representation of the body is developed, continuously updated, and maintained throughout life using visual calibration of somatosensory input (Taylor-Clarke et al. 2004; Medina and Coslett 2010). Large fibre proprioception from mechanoreceptors in the joints and muscles are particularly important, along with large fibre inputs from the skin, to the maintenance of a body representation (Paillard 1999; Longo and Haggard 2010; ter Horst et al. 2012; Cardinali et al. 2016). These somatic inputs are continuously calibrated by vision (Miall and Haggard 1995; DiZio and Lackner 2000) and also dynamically updated through corollary discharge of motor commands used to predict reafference (Ghez et al. 1995; Sarlegna et al. 2010; Miall et al. 2018).

Two influential forms of body representation are the body image and body schema (Paillard 1999). The body image involves perceptions, mental representations, beliefs, and attitudes towards the body. Though encompassing social and cultural factors, it also includes the ability to think about and make conscious decisions on the shape, size, and location of body parts. In contrast, the body schema is defined as a representation of the positions and movements of the body and its parts in space and which is more directly involved in sensory–motor control, including planning of actions which does not reach consciousness (Gallagher 1986). Body image and body schema are not independent but instead reciprocally influence each other (Gallagher and Cole 1995). These are both, of course, conceptual ways of looking at how peripheral feedback which is perceived, and that which is not, relate to action. The concepts cannot be easily associated with identifiable, neuroscientific areas or networks within the brain.

Here, we have tried to determine how the absence of peripherally originating somatosensory information affects both conscious perception and subconscious representation of the body through perceptual and action-based tasks. We presume that tasks of perception of size and position usually require conscious attention and so involve conceptually the body image, whereas those requiring a rapid response may uncover more automatic, body schema, mechanisms. Though these are empirical observations, the body schema/body image conceptual distinction may have some useful explanatory role in interpreting our results more widely, since it has wide currency.

Acquired somatosensory loss of peripheral inputs from the body is thought to involve an auto-immune triggered response, resulting in the loss of large myelinated sensory fibres and leaving the subject without the sense of touch or proprioception (Cole 1995). In rare cases, the loss is so massive that sensation is lost from the mouth or neck down to the feet; clinical tests suggest that motor and small sensory fibre function is unaffected. Onset has been in adulthood, after these subjects have acquired normal somatosensory representations and a normal motor repertoire.

Gallagher and Cole (1995) discussed one such subject, IW, in relation to body image and schema. They suggested that while neurologically normal subjects used their body schema for most movement control, and body image for conscious reporting, IW used more conscious processes to control most actions, and so, in simple terms, might be said to employ his body image to replace in part his reduced access to a body schema. Indeed, ter Horst et al. (2012) found that IW's motor imagery processes, based on the body schema, were impaired, whereas his visual imagery processes were enhanced compared to controls. Paillard (1999) discussed another case, GL, who like IW lost sensation as an adult, and suggests she “resorts to a memorised visual representation of her body and the relative positions of their mobile segments;” in other words, a body image.

Recently, a woman (KS) was identified whose absence of somatosensory afferents is congenital, and who therefore has never had somatic sensation, and thus in whom movement guidance and perception appears to be exclusively of visual origin. It should be noted that KS lacks small as well as large diameter sensory fibres from her entire body and head.

We aimed to compare various aspects of body awareness and action control in these two rare individuals. First, we asked them to report on the shape and size of their hands. Longo and Haggard (2010) report that normal participant’s representation of their hand is consistently distorted, possibly reflecting in part a distorted somatosensory representation (Penfield and Boldrey 1937) and differences in sensory resolution across the limb (Cody et al. 2008). Given the chronic and congenital loss of somatosensation in IW and KS, we expected to see differences in accuracy and distortion compared with controls and, possibly, each other.

Next, we investigated the accuracy of responses in a reach-distance judgement task to test the representation of arm length (Heft 1993). These judgements combine information from visual input and a body schema. Neurologically normal adults typically slightly overestimate reach (Carello et al. 1989; Bootsma et al. 1992; Heft 1993; Rochat and Wraga 1997; Mark et al. 1997; Leclere et al. 2019). They are more accurate when the reaching task is unattended, performed as a secondary task, suggesting that the overestimation is caused by conscious reflection (Heft 1993). Without somatosensory input, and with a minimal body-schema (Gallagher and Cole 1995; Proske and Gandevia 2012), we hypothesised that IW would overestimate reach distance. Indeed, Coello and Delevoye-Turrell (2007) have reported that GL (with adult-onset deafferentation) overestimates her reach more than controls. Instead, KS, due to her congenital deafferentation, might show greater accuracy, if she has developed an entirely visually based form of body representation. An alternative is that KS might not have developed a normal sense of distance; accuracy in reach judgements improves only gradually during childhood (Gabbard et al. 2007). She reports poor depth perception and, for example, finds judging the differences in distance between people in a room problematic. This may reflect her sensory deafferentation per se, or the fact that she has lived from a wheelchair and not explored extra-personal space as others do. IW reports normal depth judgement.

Finally, we assessed the influence of peri-personal space on spatial attention, which is a fully subconscious process. In the neurologically intact, detection of visual or auditory stimuli is facilitated when targets are presented close to the perceived location of the hands (Reed et al. 2006), and targets near the hand are represented with greater visual resolution than far targets (Brown et al. 2015). These facilitatory effects are only seen in peri-personal space, and do not extend beyond normal reach. Thus, a central body representation can be revealed by its influence on spatial attention. As with estimates of limb length or hand shape, there is normally an integration of visual and proprioceptive signals to form the representation of peri-personal space (Macaluso and Maravita 2010). Hence, differences in reaction times that depend on target-to-hand distance are seen in normal controls even without direct vision of the hand. In fact, the use of tools that extend reach also extends the range of these attentional effects (Maravita et al. 2003), consistent with the idea that body representations are quite dynamic. We hypothesised that the peri-personal modulatory effect might be exaggerated in deafferented participants as long as the hand is visible, because of their high reliance on visual coding of their body position, but may be diminished or absent when the hand is hidden from direct view. And, as with the other experiments, we predicted that IW might exhibit a maintained, if somewhat degraded, influence from his early somatosensory experience on spatial attention, whereas KS would be entirely visually dependent.

## General methods

### Participants

Two deafferented people were tested; we refer to them as ‘test’ participants. At the time of testing, IW was a 66-year-old male with an acquired form of the condition which occurred when he was 19 (Cole 1995). He has no sense of light touch nor movement/position sense from below a level at the collar line anteriorly and extending to the top of the head posteriorly (C3 spinal level). Temperature and pain perception are clinically intact, as is motor nerve function, assessed by nerve conduction studies and EMG (Cole and Katifi 1991; Cole and Sedgwick 1992). At the time of testing, KS was 40 years old. She has a congenital absence of all somatosensory fibres over her whole body. Multiple modes of testing–nerve conduction, biopsy, and evoked potentials as well as neurological testing confirm that KS has no myelinated fibres. Neurological exam reveals that KS has no superficial small fibres over the body and head (P Mason, FA Axelrod, AT Reder, unpublished observations /personal communication). Further details of her condition will be published in due course.

Because of the age difference of the two test participants, two separate groups of control participants were recruited. IW was matched with seven controls with a mean age of 67.4 years (SD = 3.6, 3 males, 4 females) and KS with seven controls of mean age of 38.4 years (SD = 3.7, 4 males, 3 females). In the absence of a priori estimates of effect sizes, we based our numbers on our prior experience of similar studies: control comparisons with IW and other deafferented participants (Ingram et al. 2000; Balslev et al. 2007a, b; Miall and Cole 2007; Miall et al. 2018, 2019). Written informed consent was obtained for each participant prior to the study which was approved by the University of Birmingham ethics board and performed in accordance with the Declaration of Helsinki.

All participants, test and control, were fluent English speakers and had normal or corrected-to-normal vision and hearing. All participants completed the ten item Edinburgh Handedness Inventory (Oldfield 1971). The two test participants are strongly left-handed with Edinburgh handedness inventory scores of − 80 (KS) and − 100 (IW). The older control group included one left-hander with a score of − 95; all other control participants were right-handed with scores of + 100.

The control participants (*n* = 14) also completed the nine-hole peg test, with the average performance time cross-referenced to standardized values (Oxford Grice et al. 2003), to ensure that they had normal hand function. The mean duration for the young group was 18.0 s (SD 1.1 s) for their dominant hand which is within the norm for 36–40 years old (males: 17.7, SD 2.1; females: 16.7, SD 1.9; t (37) < 1.65, *p* > 0.11). For the older group, the mean duration was 18.05, SD 0.62 s which was faster than the norm for male 66–70 years old (mean 21.2 s, SD 3.3; t (19) = 2.54, *p* = 0.020) but not different from the female 66–70 year norm (19.9 s, SD 3.2, t (36) = 1.6, *p* = 0.125).

IW and KS completed the ABILHAND questionnaire (Vandervelde et al. 2010). Analysis was performed on http://www.rehab-scales.org against the calibration for patients with neuromuscular disorders. KS shows considerable impairment in everyday hand use, with a raw score of 10 (answering 14 out of 18 items) resulting in an overall ability measure of − 0.9 logits (SE 0.5 logits); normal adults would have a raw score of 36, and an ability measure of 6.4 logits (SE 1.7). KS’s low score may reflect musculoskeletal as well as neurological deficits in distal fine motor skills, and are consistent with her infrequent use of her digits, particularly the lateral digits (middle, ring, and little fingers). IW’s profile is closer to the normal, with a raw score of 34 (across all 18 items), and an ability measure of + 4.2 (SE 0.8) (Miall et al. 2019).

In addition to these two test participants and their age-matched control groups, each experiment was piloted on groups of 25–35 undergraduate students, aged 19–21. These pilot results are only mentioned in brief, where appropriate.

## Experiment 1A: hand template matching

### Protocol

**Fig. 1:**
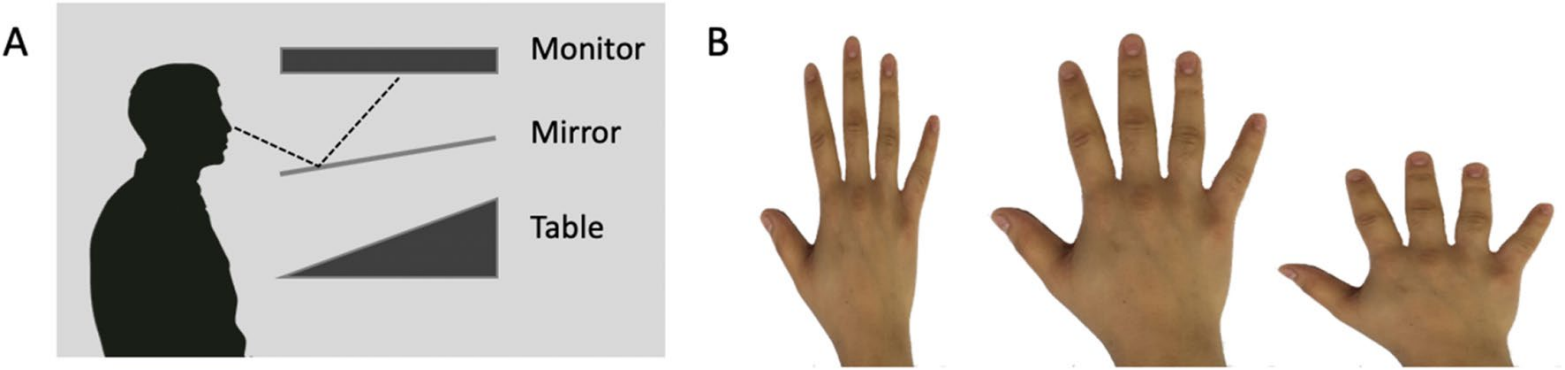
The set-up for Experiment 1(a). Participants viewed 15 images of their own hand, presented in a numbered 3 × 5 grid, with each image distorted vertically or horizontally by up to ± 35% (as shown here with left: 35% reduction of width, middle: no distortion, and right: 35% reduction of length); the participant verbally reported which image was closest in shape to their own unseen hand

This task is modelled on the methods of Kammers et al. (2009). In our task, participants sat in front of a sloped table, aligned with their body midline, with both hands comfortably held on their lap and out of view (Fig. 1). A mirror blocked direct vision of the table and reflected the image of a large flat-screen monitor (Apple Cinema HD), such that the virtual image appeared on the occluded table surface. Viewing angle was approximately 70 degrees to the surface. On each trial, 15 modified versions of a photograph of the back of the participant’s own hand were displayed simultaneously within a virtual grid of 5 × 3 numbered positions on the screen; the screen had a plain white background and the grid positions had no visible borders. Each cell was 8.7 cm wide and 9.6 cm in height. The photographs were edited to have a plain white background, and were taken of the hand with the fewest obvious identifying features; where necessary, jewellery was removed and any remaining features were edited out. This “master image” was left–right reversed as necessary to match the dominant or non-dominant hand being tested. For each trial, the master image was then reduced by 0, 5, 10, 15, 20, 25, 30, or 35% in width (or separately in length) to give 15 unique shapes (1 unchanged; 7 narrower and 7 shorter, ranging from a length/width ratio of 0.65–1.35; see Fig. 1b) simultaneously presented on the screen, in a pseudorandom order. The participant verbally reported which photograph they judged to be closest to their own hand shape, by reporting its number on the screen. This task was not timed. Control and test participants performed two blocks of 8 trials, first judging their non-dominant hand, and then their dominant hand.

### Analysis

The mean and SD of the length/width ratio chosen across eight trials was recorded, and the control data compared with ANOVA and one-sample *t* tests against an expected ratio of 1.0. To compare each test participant with their respective control group, *t* scores (instead of *z* scores, because of the small sample size) were calculated.

## Results: experiment 1A

**Fig. 2:**
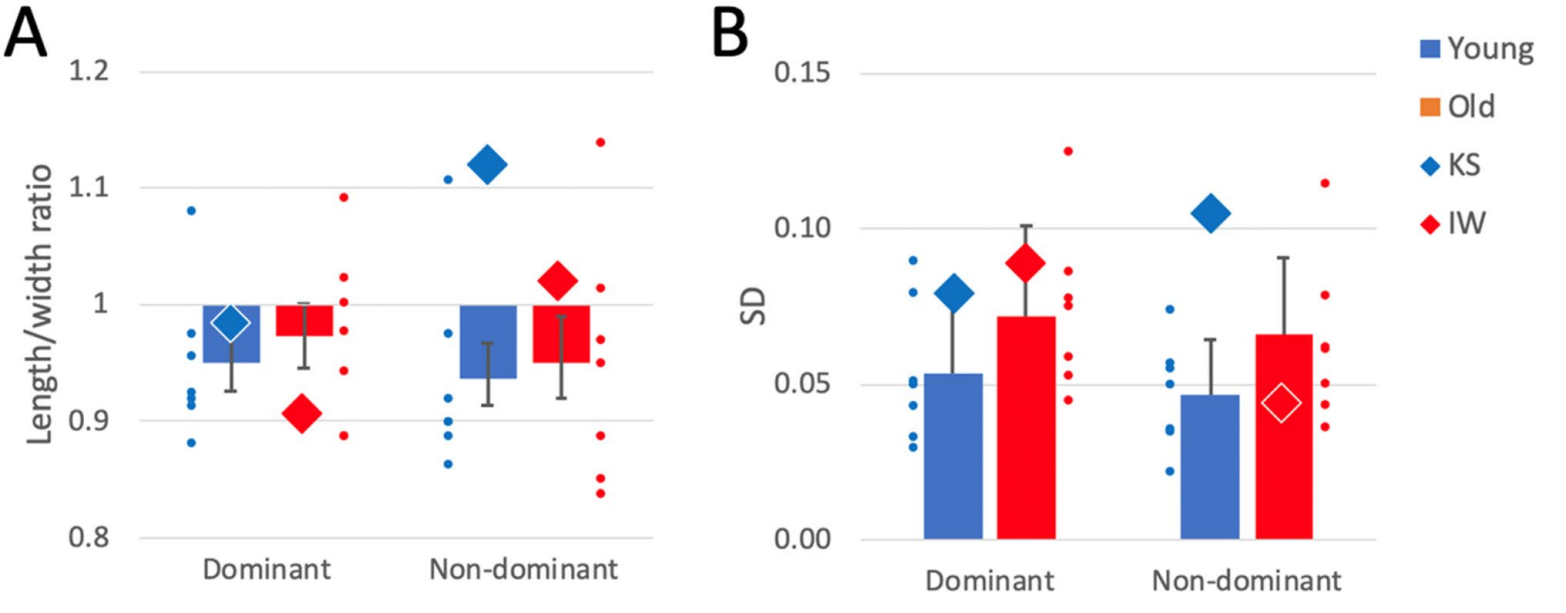
Mean length/width ratios **a** for the young (blue bar) and older (red bar) control groups (*n* = 7, mean + 1SEM) and for KS (blue diamonds) and IW (red diamonds). Panel B shows the within-participant standard deviation across 8 trials (*n* = 7, mean + 1 SEM). The small blue and red dots are the individual data for the younger and older controls, respectively; other conventions as in panel A

We first tested for any significant difference between the two control groups for the images chosen as most similar in shape to their dominant and non-dominant hand, with a mixed ANOVA (age group × hand). There was no significant effect for either age [*F*(1,12) = 0.14, *p* = 0.72] or hand [*F*(1,12) = 2.43, *p* = 0.15; Fig. 2a]; nor was the interaction significant. The standard deviation of choices was relatively small (Fig. 2b). Using one-sample within-participant *t* tests, we found that five of the seven younger controls selected length to width ratios significantly less than the veridical value of 1.0 for both dominant and non-dominant hands, while one participant chose a ratio significantly greater than 1.0; for the older control group these participant numbers were three and one, respectively; the choices of the remaining (4/14) participants were not significantly different from 1.0.

In other words, most control participants tended to reliably select images slightly wider and shorter than their own hands. Combining across both hands, and averaging across all 14 controls, the overall mean length/width ratio was 0.95, significantly below the veridical value of 1.0 [*t* (13) = − 2.31, *p* = 0.04].

We then tested the difference between IW’s and KS’s selected length/width ratio and their age-matched control group, by calculating *t* scores. For the dominant hand, the mean values were again less than unity but the differences from controls were not significant (IW: mean 0.91, *t* (6) = − 1.62, *p* = 0.16; KS: mean 0.98, *t* (6) = 1.38, *p* = 0.22). However for their non-dominant hand, both chose image ratios that were greater than unity (IW: 1.02; KS: 1.12; Fig. 2a, diamonds), with differences from controls that were a trend for IW [*t* (6) = 2.13, *p* = 0.077] and significant for KS [*t* (6) = 5.90, *p* = 0.001].

## Experiment 1B: hand-mapping task

### Protocol

**Fig. 3:**
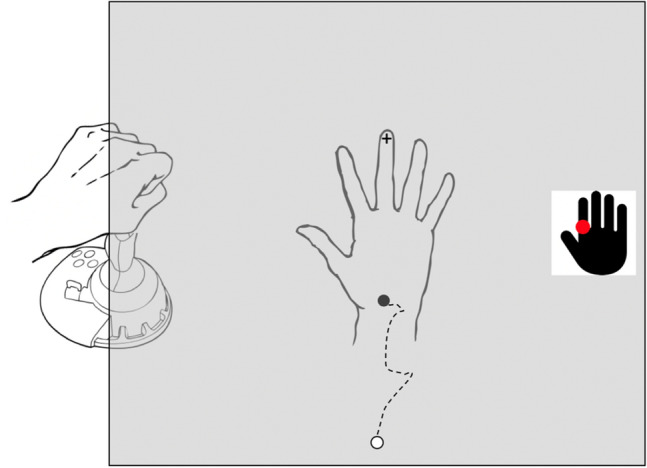
A schematic of the hand configuration task, Experiment 1b. Participants rested their test hand (in this case the right hand) on a sloped surface beneath an occluding mirror (see Fig. 1a), with the middle finger touching a small raised bump ( +). On each trial, an icon appeared at the right side of the screen with a target location marked (red dot). Control participants then used their other hand to move a cursor (black dot) that appeared at the bottom of the screen, to the perceived landmark on their unseen hand and, when ready, pressed the joystick trigger button. IW and KS controlled the cursor by verbally instructing the experimenter, who manipulated the joystick on their behalf

Procedures for this task are similar to those of Longo and Haggard (2010), in which participants use a mouse or pointer to locate landmarks on their unseen hand. Participants placed their test hand palm-down on a sloped table (Fig. 1a) aligned on the body midline, with their hand flat and fingers comfortably spread (Fig. 3). A mirror occluded a view of the hand and reflected a horizontal computer display screen, such that the images appeared in the plane of the hand. Participants were able to look below the mirror to position their hand with the tip of the middle finger resting on a small raised disk (felt by the controls and visible to IW and KS). IW and KS needed to reposition their hand intermittently when informed that it was slipping out of position.

On each trial, participants were visually cued which landmark to localise, with a small dot shown on a stylistic icon of a hand, displayed towards the edge of the screen. The experimenter also verbally described the target (e.g., “tip of the index finger”, “knuckle of the thumb”, etc.). To our surprise, participant KS (a college and law school graduate) stated that she did not know the names of the fingers, and required a brief training session to ensure she could identify them by name, and she occasionally asked for reminders. This might reflect her limited dextrous ability and experience. Control participants used a Microsoft Sidewinder joystick held with their non-dominant hand to control the motion of a cursor on the screen that appeared in each trial at the bottom of the screen in the midline; deviation of the joystick from its central sprung position controlled the speed of cursor motion, with the gain of the response adjusted for each individual so that they could easily and smoothly control the motion. They positioned the cursor (2 mm diameter), without any time limit, and then pressed the joystick trigger to indicate their perceived location of the cued landmark on the occluded hand. The two test participants found the joystick difficult to use, without direct vision of their hand on the handle. They therefore gave verbal instructions to the experimenter (“up”, “left” etc.), who controlled the joystick and made the trigger response when instructed to do so on their behalf.

Fifteen landmarks on the dominant hand were mapped by most control participants: the proximal and middle knuckles (i.e., centre of the knuckle at the base of each finger and at the middle joint) and the fingertips (i.e., most distal point) of each digit; only ten landmarks were used for three control participants, excluding the middle joint. Each control participant performed four blocks of 15 trials, with landmarks presented in a pseudorandom order.

The two test participants IW and KS performed four blocks of 20 trials, across 3 days of testing, two with the left hand and two with the right hand. Because of the additional time needed for them to verbally direct placement of the cursor, each block tested only ten landmarks; the proximal knuckle and the fingertip for each of the five digits, each landmark tested twice in each block. At the start of each block and occasionally within a block, IW and KS visually inspected their hand. The two test participants also performed one block of 20 trials in which they were allowed to inspect the hand on every trial, and a further block where their hand remained on their lap, and they reported the positions of the landmarks as if the hand was in position on the table. These extra tests were only conducted with the dominant left hand.

### Analysis

The mean position of each location selected for each hand landmark was calculated after any individual datum more than 5 cm from the mean was excluded. Across all 14 controls, 25/840 data points (~ 3%) were excluded, with a maximum of 10 in one participant. Only one datum each was excluded for KS and for IW. However, for all control participants, there was a maximum of one datum excluded for any landmark position, leaving at least three valid data. Note that with the exception of the middle fingertip, located on a marked position, the true position of each landmark depends both on hand shape and size, but also on the spread of the fingers. The participants were told to comfortably spread their fingers, and so adopted quite repeatable postures. However, we report digit lengths (the distance between the mean reported location of the primary knuckle and fingertip of each digit) and hand width (the distance between the primary knuckle of the index and little finger), as these measures are unaffected by spread of the fingers. To compare each test participant with their respective control group, condition-specific *Q*’ scores were calculated (equivalent to case–control *t* scores) and across-condition *Q*’ scores used to test if the variations in the case–control differences across the factors (hand and digit) were significant (Michael 2007; Renault et al. 2018).

## Results: experiment 1B

**Fig. 4:**
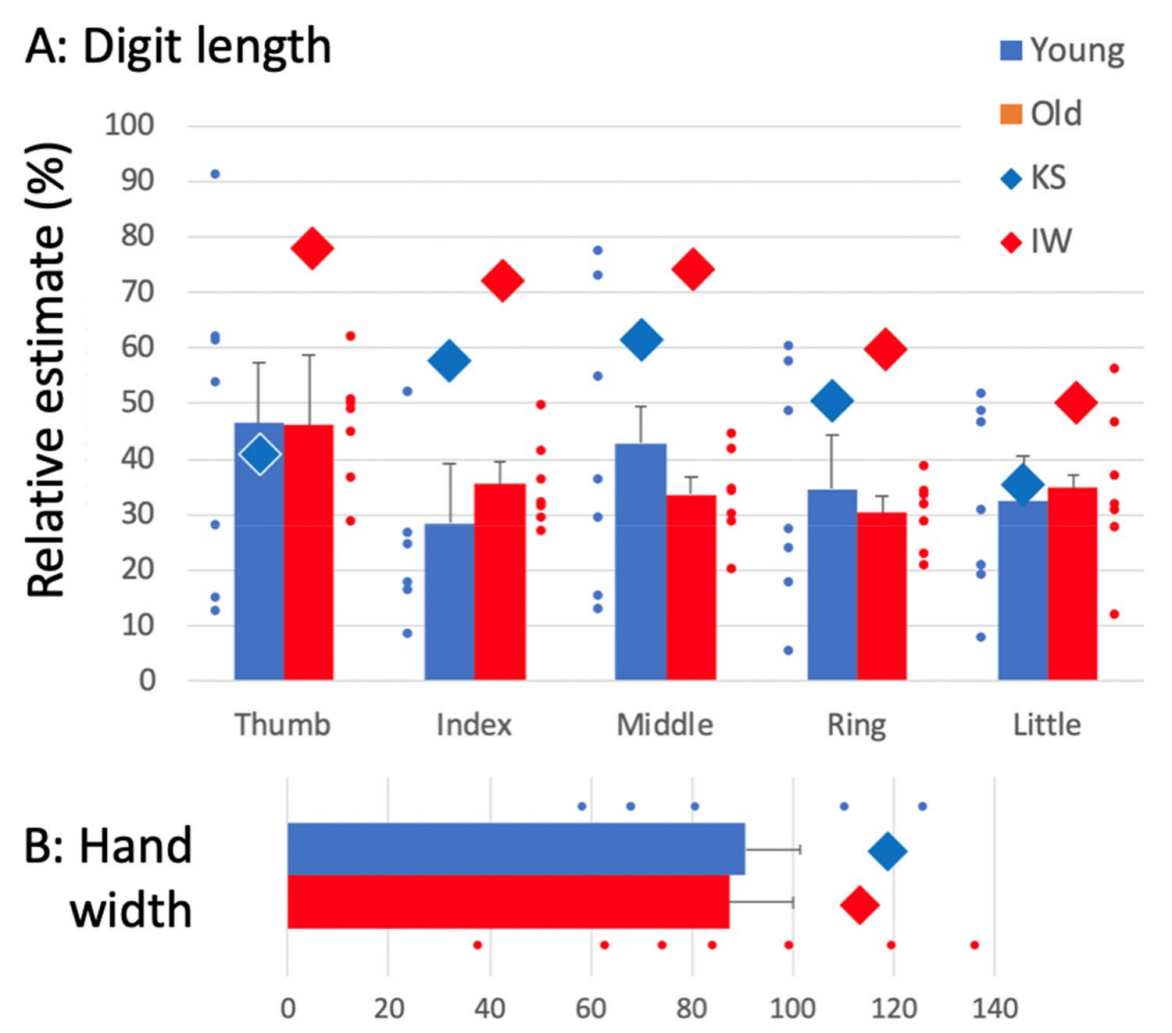
Estimated digit lengths **a** and hand width **b** as percentages of the actual. **a**: Vertical bars represent the mean distance (+ 1 SEM for the younger and older control groups, each *n* = 7) between the estimated position of the proximal knuckle and the fingertip, for each of the five digits; blue bars are for the younger control group, red for the older group. The small blue and red dots are the individual data for the younger and older controls, respectively. The large diamonds are means for KS and IW, averaged over four sessions. **b**: Horizontal bars represent the % distance between the estimated proximal knuckle positions of the index and little fingers. Symbols as in panel A

The distances between the perceived positions of each landmark on the unseen hand showed that control participants tended to underestimate the mean width of their hand (the separation between the proximal knuckles of the index and little fingers). As a percentage of their actual hand width, the mean estimate was slightly below unity: 90 and 88% for both younger and older control groups, respectively (Fig. 4b). However, controls grossly underestimated the length of each digit, reporting lengths only 37 and 36% of the actual, for the younger and older controls, respectively (averaged across all participants in each group, and across all five digits).

**Fig. 5:**
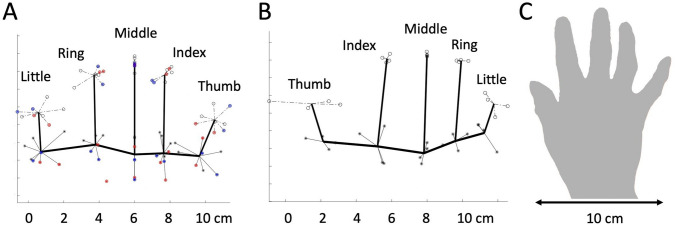
KS’s reported hand maps. **a**: Left hand and **b**: right hand. The thick black lines join the mean estimated position of the proximal knuckles, and the tip of each digit, for her left (dominant) and right (non-dominant) hands. The data points are the reported positions across multiple sessions, each linked to the spatial mean for that landmark. For the left hand, data were collected over four sessions. In one session, the hand was held on the lap (red data); this session produced the only outlier (a location reported > 2 cm from the cluster mean), which was thus discounted. This red datum is shown unlinked from the cluster mean for the proximal knuckle of her ring finger. In another session, KS viewed her hand in situ, between each trial, while each landmark position was reported when view of the hand was blocked (blue data). Note the high lateral accuracy for the middle fingertip which was held at a constant midline location on the table. **c**: KS’s right hand, at a reduced scale compared to panel B. In all panels, the horizontal and vertical scales are equal

**Fig. 6:**
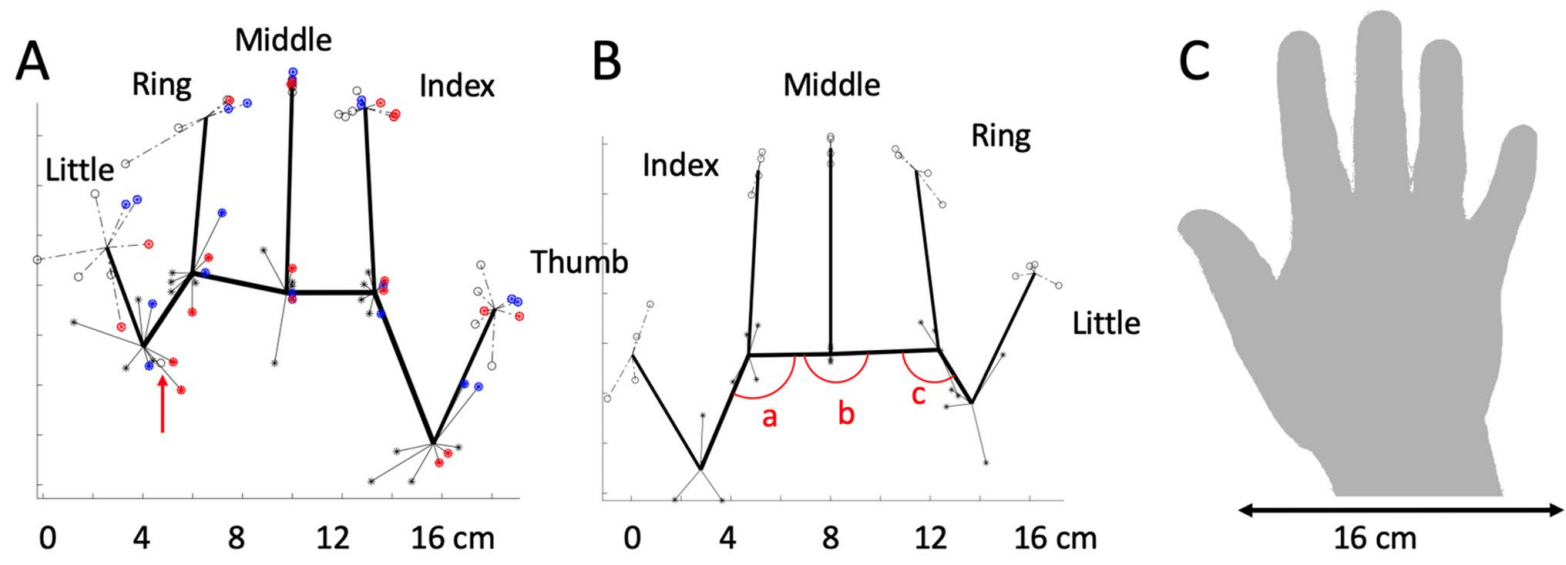
IW’s hand maps. Format is as in Fig. 5. There is one outlying data point (red arrow, panel A), where one location for the fingertip of the left little finger was reported near to the location of the proximal knuckle; this datum is shown unlinked from the fingertip cluster mean and was excluded from the mean. The red arcs (panel B, labelled **a**, **b**, **c**) represent the knuckle angles reported in the main text

IW and KS were tested in four sessions, twice reporting with the hand-held static beneath the occluding mirror (as it was for the controls), once when they viewed the hand beneath the mirror in between successive trials, and once when the hand was on the lap, out of view and remote from the visual display screen. Although the number of trials in these latter two conditions was small, there were no dramatic differences in the data from each trial condition (see red and blue data in Figs. 5a, 6a). We therefore collapsed data from across all 4 sessions for the left dominant hand. For the right (non-dominant) hand, we collapsed across two sessions tested with the hand static, as it was for the controls. The lack of a difference in results when the test participants had their hand on their lap or on the surface provides dramatic evidence that they derived no benefit from the placement of their hand.

KS mis-located her hand landmarks, such that we could infer she overestimated hand width by 19% (Fig. 4b, blue dot), significantly more than her controls did (*Q*(6)’ = 2.20, *p* = 0.014). However, like controls, she substantially underestimated her digit lengths, with an average across the five digits of 49% (Fig. 4a). Nonetheless, she tended to be closer to veridical (100%) than the younger control group (37%). For her index, middle, and ring fingers, these differences were significant [(*Q*’6)’ > 1.71, *p* < 0.043)]; for the thumb and little finger, they were not significant [(*Q*’6)’ < 0.47, *p* > 0.32)]. Figure 5a shows the configuration of the positions which she reported, and the mean position for each landmark. It is apparent that she exaggerated her hand width and underestimated the digit lengths.

IW was the most accurate of all participants, young or old, with an overall average digit length estimate that was 66.8% of the actual (red dots, Fig. 4), and a hand width overestimate of 13%. All differences from his control group were significant [*Q*(6)’ > 1.82, *p* < 0.034), and the pattern across the six measures was also significantly different [Q(5)’ = 17.22, *p* = 0.004; Fig. 6].

The estimates by KS and IW of their right, non-dominant, hands were very similar to their dominant (left hand) estimates, but were marginally closer to veridical (100%) with slightly less overestimation of the width and slightly less underestimation of digit lengths (53.4 and 71.6%, respectively, averaging across all digits).

One striking feature is that KS estimated the primary knuckles of her thumb and little finger to be about level with the other knuckles (i.e., all five knuckles are on a line, Fig. 5), whereas, in reality, they—and particularly the thumb base—are closer to the wrist [see, for example, Fig. 2e of Longo and Haggard (2010)]. We quantified this by calculating the angles between the neighbouring triplets of knuckle positions (thumb–index–middle, index–middle–ring, and middle–ring–little fingers; see the arcs indicated on Fig. 6). For KS, her “thumb angle” (arc a) was significantly flatter than the controls for both her hands [144 and 176 degrees, left and right, respectively, vs 95 degrees ± 11 SEM: *Q*’(6) > 2.97, *p* < 0.0015]. The angle across the middle finger knuckle (arc b) was not different from the controls for either hand [*Q*’ < 1.17, *p* > 0.12]. Her “little finger angle” (arc c) was somewhat flatter (168 and 174 degrees vs 150 ± 18), but these differences were not significant [*Q*’(6) < 1.2, *p* > 0.11). For IW, his thumb angles were not significantly different from the controls (111 and 114 degrees vs 117 ± 13; *Q*’(6) > − 0.47, *p* > 0.3). The angle across the left middle finger knuckle was not different from the controls (*Q*’ = 1.13, *p* = 0.13), while it was flatter for the right hand (*Q*’ = 2.02, *p* = 0.22). Finally, his little finger angle was more acute for both hands (112 and 119 degrees vs 148 ± 10.6, *Q*’(6) < − 2.26, *p* < 0.012).

## Experiment 2: reach-distance estimation

### Protocol

This experiment was based on the design used by Carello et al. (1989) and Gabbard et al. (2005). Participants sat at a table covered with a large featureless white card surface 1 m wide by 1.5 m in depth; side walls 0.3 m high blocked view of immediate landmarks. A single line was drawn on the surface in the participant’s midline, and extending the full 1.5 m depth of the card. Participants were asked to reach out once along this line with their dominant arm, to their maximal distance with fingers extended, but without leaning forwards in their chair (Heft 1993). This distance was noted and used as the central location for subsequent test trials. Because of the challenge that IW and KS have in moving without vision, all participants made this initial reach movement with full vision of the table and the surrounds.

On each trial, a large cardboard screen was placed vertically, across the table, blocking the participant’s view of all but the closest 10–15 cm of the surface. The investigator placed a penny coin on the table, along the midline, at a randomized distance from the participant’s maximal reach distance. The cardboard was then lifted and the participant instructed to respond immediately with a verbal yes/no as to whether they thought that they could reach the coin. To encourage rapid responses, the participants were instructed to speak into a microphone and were told their reaction times were being monitored; in practice, these recordings were too noisy to provide unambiguous response time measurements.

Both deafferented participants were presented with targets at nine distances around their maximum reaching distance: for IW and KS: − 9, − 6, − 3, 0, 3, 6, 9, 18, and 50 cm around their individual reaching distance. IW and KS were tested on 2 successive days, with 45 trials on each day, first making judgements for their dominant arm and then for their non-dominant arm; actual reach distance was measured only the once, with dominant hand. For the controls, we reduced the range to -6, -3, 0, 3, 6, 9, 12, 18, and 24 cm around their actual reach distance, and they made reach judgements for their dominant arm only, across 45 trials (5 at each distance).

### Analysis

Reach judgements were scored (0 = reachable, 1 = unreachable) and the responses were converted to a proportional score, across the five repeat measurements at the nine distances. The tested distances were converted to a percentage of the arm length for each participant, and a logistic curve was then fitted using the Matlab function *glmfit*. After initial curve estimation, any residuals more than 2 SD away from the curve were excluded. After removal of these outliers, curves were refitted to estimate each participant’s bias point (the point of subjective equivalence, 50%) and the JND (the interval between 25 and 75% of the curve). In total, four data points were excluded (2.5% of the total), one from one young control, one each from two older controls, and one from KS’s non-dominant arm test.

Comparisons between the test participants and their age-matched controls were made with t scores as in Miall et al. (2018, 2019).

## Results: experiment 2

**Fig. 7:**
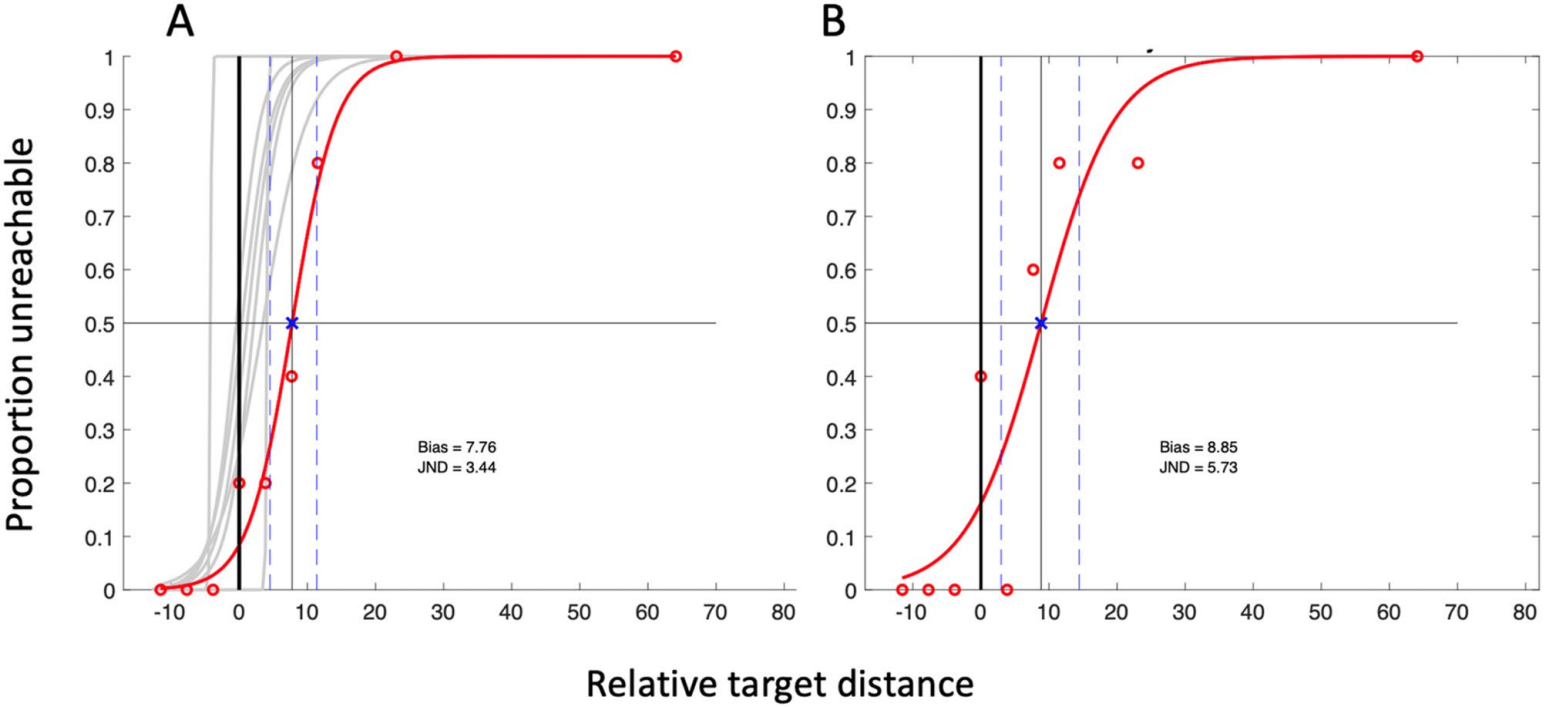
The individual reach-estimate psychometric fits of the older control group (grey curves) and IW (red curve and data points) for the dominant arm **a** and, for IW only, the non-dominant arm **b**. The horizontal axis is target distance relative to actual reach distance (thick black vertical line), presented as percentage of arm length. The thin black vertical line and blue x mark IW’s bias (50:50 point); the bias points for the controls not shown. The two blue dashed lines surrounding IW’s bias point are the 25 and 75% points of the curve; the interval between these is his JND

**Fig. 8:**
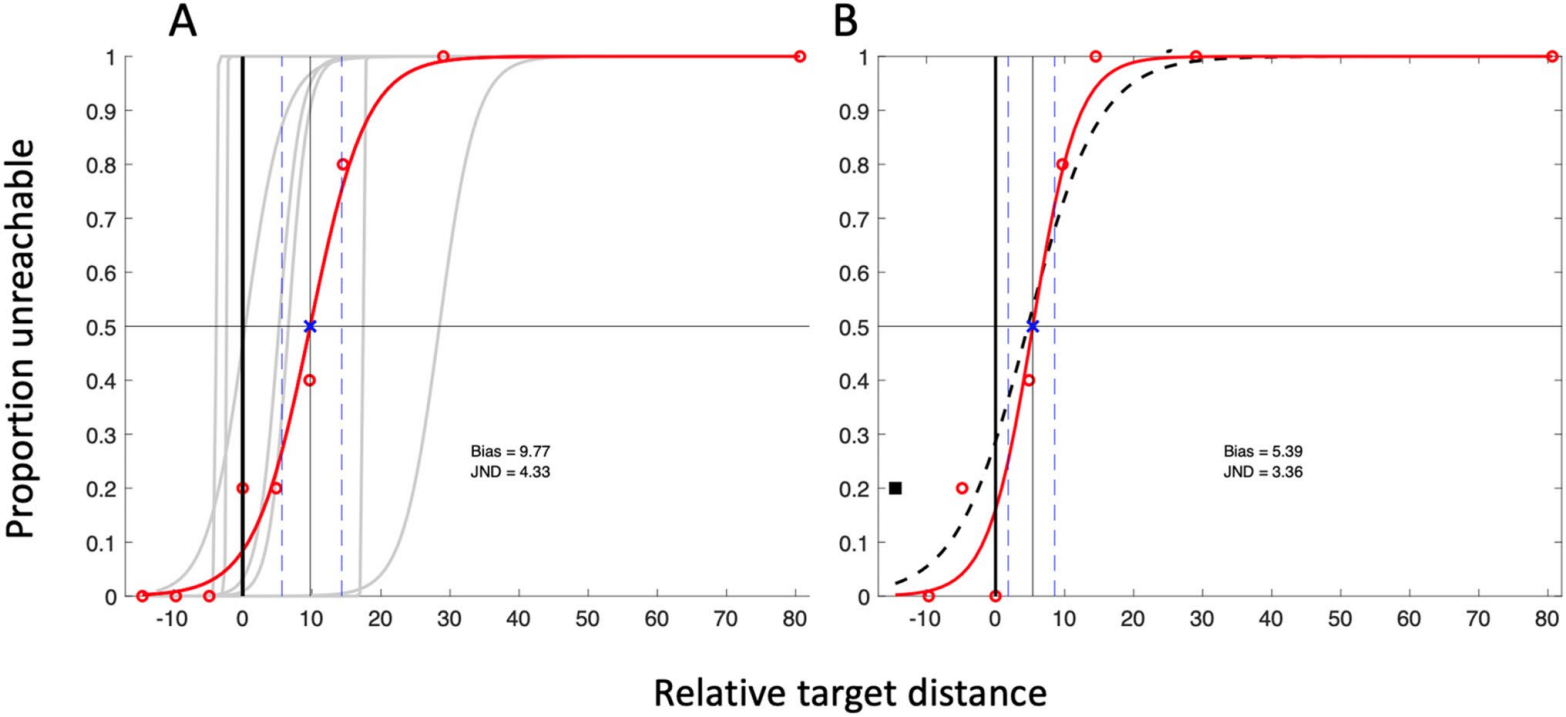
The psychometric fits for the younger control group and for KS, in the same format as in Fig. 7. In panel B, the dashed black curve is the initial logistic fit; the left-most data point (black square) was identified as an outlier and removed before the final fit (red curve) was achieved

We extracted the bias point from each psychometric curve (Figs. 7, 8) to estimate reach distance, and used the JND as a measure of participants’ precision in their decisions. The young and older control groups showed the reported tendency to overestimate their reach distance, by an average of 7.4% (SD 11.7%, one-tailed, one-sample *t* test *t* (6) = 1.67, *p* = 0.072) and 1.5% (SD 1.7%, *t *(6) = 2.36, *p* = 0.028) of their arm length, respectively. The two control groups were not significantly different [*t* (12) = 1.31; *p* = 0.236]. The JND of their judgements were also not significantly different (1.47 (SD 1.31) vs 1.83 (SD 1.25); *t* (12) = 0.53, *p* = 0.606); nor were their arm lengths different (mean 71.3 cm (SD 3.29) vs 73.4 cm (SD 2.86); *t* (12) = 1.61, *p* = 0.135).

Both IW and KS also overestimated their reach distance (by 7.8 and 9.8%, respectively, Figs. 7a, 8a), each a greater overshoot than their respective control group: this difference was highly significant for IW (*t* score = 9.51, *p* < 0.0001), but not for KS (*t* score = 0.53, *p* = 0.26).

However, unexpectedly high variance for the bias estimates from the younger control group (mean age 38.4) confounded this comparison for KS. In a related study, 31 undergraduate adults (“UG”, age range 19–21, mean age 19.7) performed the same reach estimation task. Their mean bias was the same magnitude as that of KS’s control group (7.9% for the UG group, compared to 7.4% for KS’s group), but the variance in the bias measures across the UG group was considerably less (SD = 4.9 vs 11.7% for KS’s group). The mean JND for this new group was 3.3% (SD = 1.72%) compared to 1.5% (SD 1.3%). Taking into account the larger group of undergraduate controls lends power to our conclusion that KS performed no differently from controls in reach estimation.

**Fig. 9:**
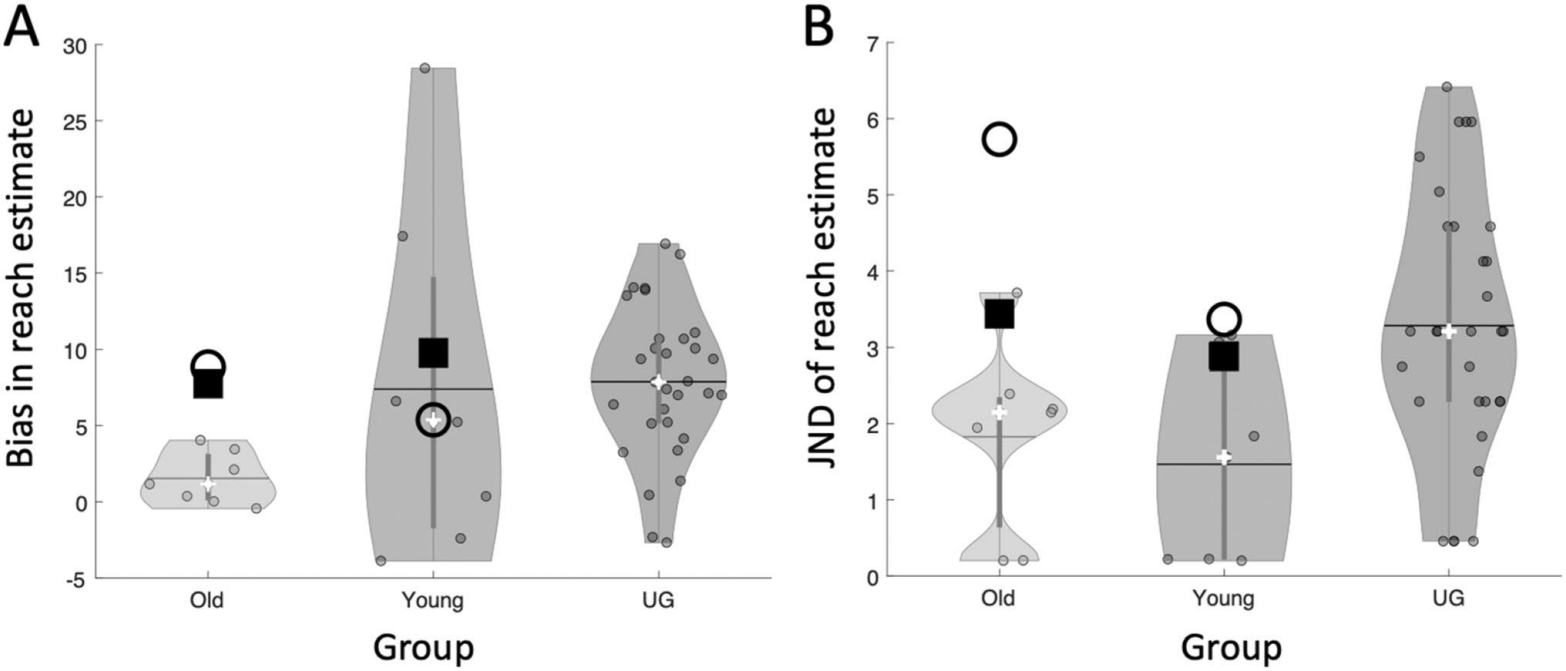
Violin plots for the control group’s distribution of reach estimation bias **a** and JND **b**; the small white crosses are the group medians; horizontal lines are the group means. The black boxes are the bias and JND estimates for the dominant arm for IW and KS (against the old and young controls, respectively); the black circles are IW and KS’s non-dominant arm estimates. The data labelled UG is from a larger group of undergraduates

The JND values were higher for both IW and KS than for the controls (for IW: 3.4%, *t* score = 3.40, *p* = 0.0072; KS: 2.9%, *t* score = 2.86, *p* = 0.0143; Fig. 9b), suggesting that they were less precise in their decisions.

Similar estimates of reach distance were taken for the non-dominant arm for IW and KS (Figs. 7b, 8b). While we do not have data from the control groups to compare, there are no reports of differences in reach estimates made in the midline, between the two arms (Fischer 2005; Gabbard et al. 2005; Linkenauger et al. 2009). For KS, the responses for dominant and non-dominant arms were very similar (Fig. 7a, b; Fig. 9). However, for IW, while bias was similar for both arms, his dominant arm JND tended towards the high end of the distribution of the older control group data, and for the non-dominant arm, it was unusually high (Fig. 9b).

## Experiment 3: visual attention task

### Protocol

**Fig. 10:**
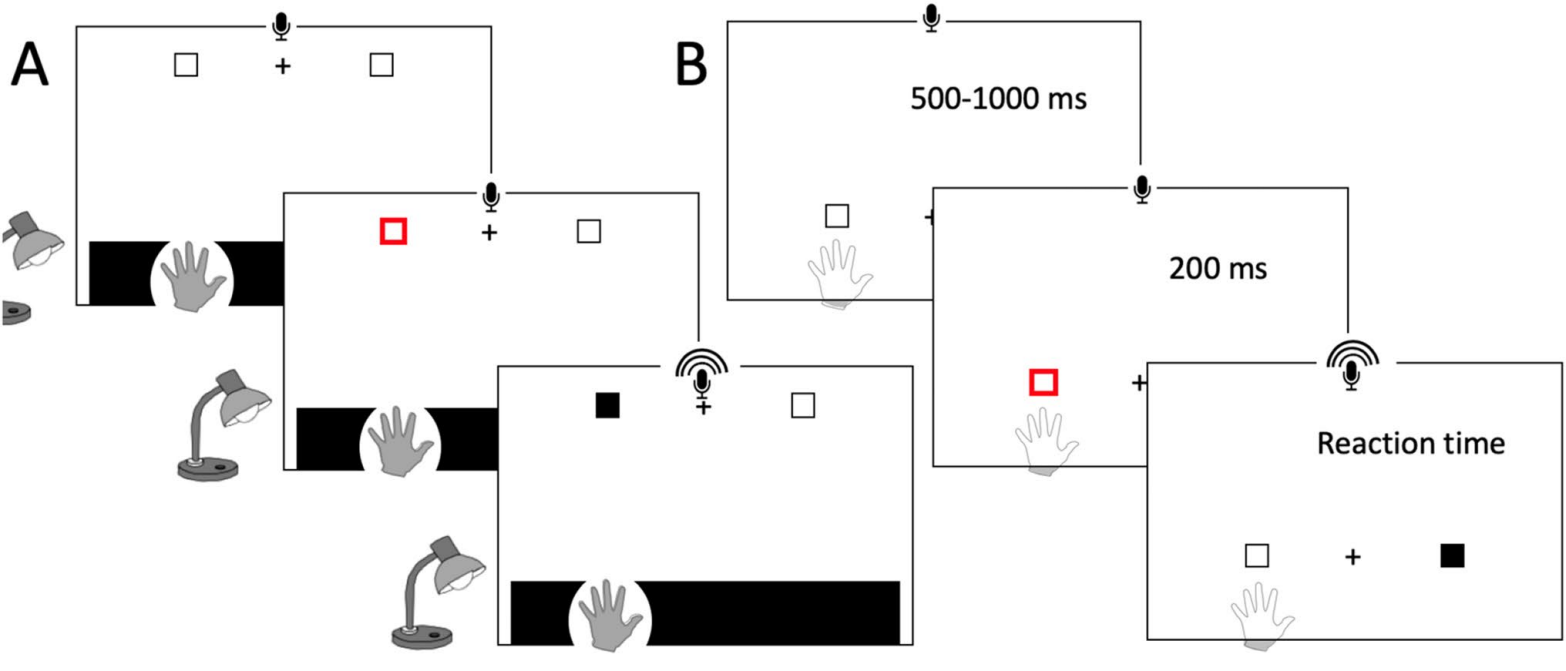
Visual attention task. The participant rested one hand on a table beneath an occluding mirror that reflected a display screen, such that targets were in the plane of the hand. Through the selective application of a sidelight, the hand was either visible **a** or not visible **b** through a black window. The outline of the hand shown in B is for display only and neither the hand nor the arm was visible in this condition. In each trial, after participants fixated on a central cross (see **a**: top panel), two placeholders would appear: placeholders were located either far **a** or near the hand **b**. One would be highlighted (red) as an attentional cue (middle panels), and then, the target would appear (black square, front panels) congruent **a** or incongruent to the cue **b**. Vocal reaction times were detected by microphone

Participants sat in front of a mirror system similar to that shown in Fig. 1, but with a horizontal semi-silvered mirror and horizontal table. The screen monitor above the mirror was used to present visual targets that appeared as virtual images in the plane of the table. A portion of the display screen could be turned black against an otherwise white background, such that the participant could see through this black “window” to directly view their hand on the table, through the semi-silvered mirror. Lighting was carefully controlled, so that with a side lamp illuminating the table surface, beneath the mirror, the hand was easily visible. Without the illumination, the reflected white background blocked view of the hand (Fig. 10). The mirror position also blocked any direct view of the arm.

At the start of each testing block, one hand was placed in line with the shoulder at a comfortable distance; hand position was controlled by opening a small black “window” in the white background. Once comfortable, the display changed to have a wide black window that spanned the full width of the display.

In each trial, a small central fixation cross was displayed for 500–1000 ms, along with two surrounding black rectangles as placeholders indicating where the target could appear. The fixation cross and the placeholders were pseudo-randomly presented near to the hand or at the far edge of the display screen, outside reach distance. This was then followed by one of the two placeholders being replaced by a red outline, acting as a Posner-like attentional cue (Posner 1980; Nougier et al. 1994) to shift attention towards the lateral target. After 200 ms, the target appeared, with 70% probability to be on the cued side and 20% probability to be on the opposite side. On the remaining 10% of trials, no target appeared. Participants were required to react to the presentation of the targets flashed onto the screen, with a verbal response (the word “target” that has a plosive onset); if they responded on the infrequent catch trials, they were reminded to only respond if the target appeared. Reaction times were logged by computer, detecting the onset of the vocalisation using a voice key. Reaction times less than 100 ms or greater than 1000 ms post-target-onset were considered invalid. All participants, control and test, performed this task with both their dominant and non-dominant hands.

### Analysis

The mean reaction times for each condition were calculated after outliers were excluded (median number of exclusions per participant: 2/320 trials, maximum for any one participant 4/320, 1.25%). Control participant data were compared with ANOVA (hand: dominant/non-dominant, vision: hand visible/occluded, target location: ipsi-/contralateral, and target distance: peri-/extra-personal). To compare each test participant with their respective control group, *Q*’-scores were calculated (equivalent to case–control *t* scores) and *Q*’-scores used to test if the variations in the case–control *Q*’ scores across the factors (vision and hand–target proximity) were significant (Michael, 2007).

## Results: experiment 3

An omnibus ANOVA showed that were no main effects for the hand (dominant vs non-dominant), and so the data were collapsed across both hands for further analysis. We found the expected differences in reaction times in the control groups, with shorter RTs in trials where the target was ipsilateral to the seen hand, in personal space. In an omnibus, five-way mixed ANOVA with factors of age group (young/old), cueing (valid/invalid), vision (seen/unseen), location (personal/extra-personal), and laterality (ipsi-/contralateral), the factors of group [*F*(1,13) = 11.31, *p* = 0.005] and cueing [*F*(1,13) = 8.61, *p* = 0.012] and an interaction between cueing, location, and laterality [*F*(1,13) = 5.15, *p* = 0.041] were significant. The group effect was driven by faster RTs in the younger cohort (459 ms, SEM 20 ms compared to 554 ms, SEM 19 ms). The significant effect of cue validity provided confidence that the paradigm was engaging spatially selective attention. The three-way interaction also supported the hypothesised effect of attentional bias proximal to the hand. There was also a near-significant four-way interaction between cueing, vision, location, and laterality [*F*(1,13) = 4.26, *p* = 0.060], such that RTs were particularly short for trials with the target near the visible hand, in peri-personal space.

**Fig. 11:**
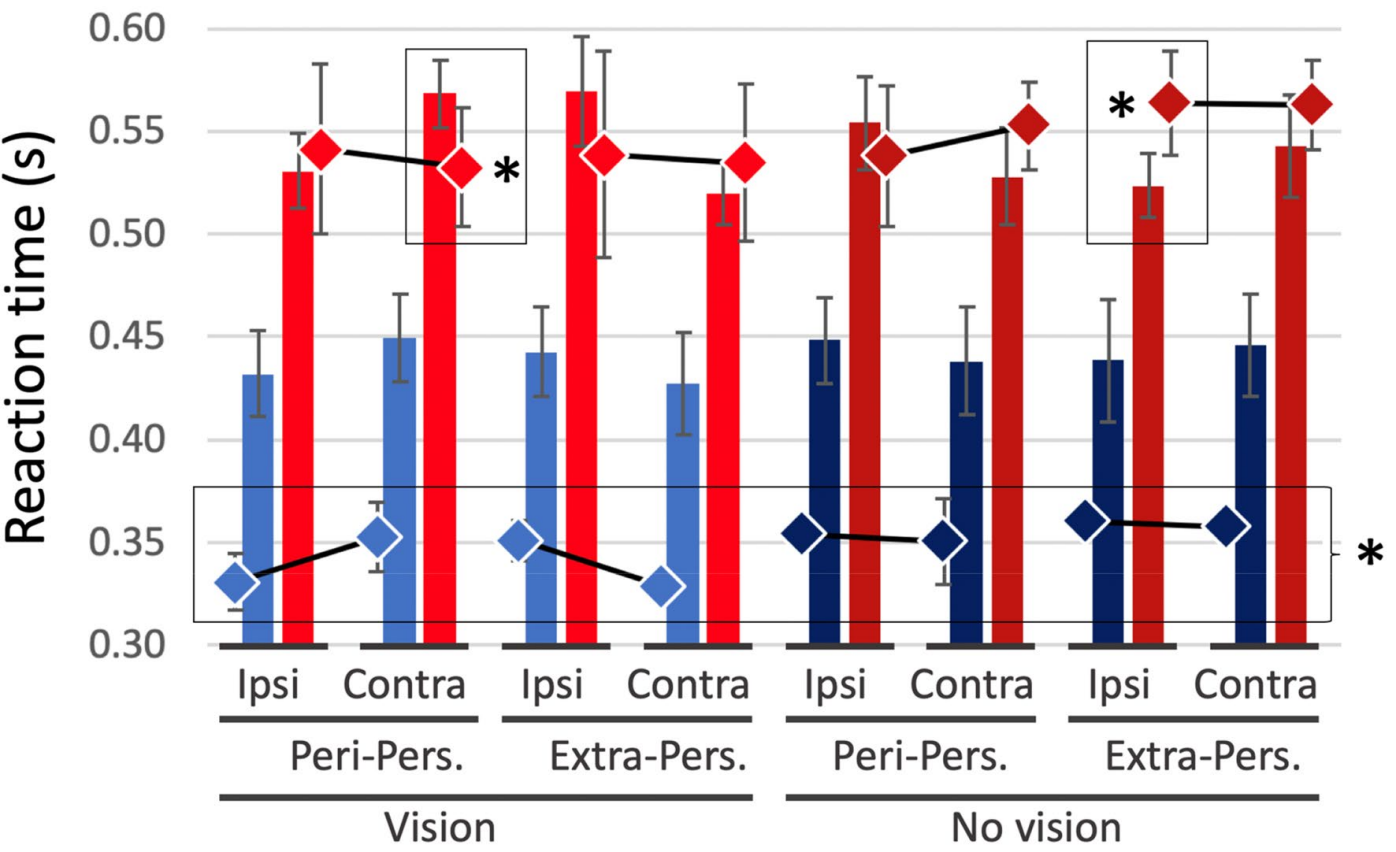
Mean reaction times for the young (blue/dark blue bars) and older control groups (red/dark red bars) for detection of targets in valid-cue trials; error bars are SEM across the group. The hand was visible (vision–light colours) or not (No Vision–dark colours); targets appeared in personal (Peri-Pers) or extra-personal space (Extra-Pers), ipsilateral (Ipsi), or contralateral (Contra) to the hand. The diamonds are the means across daily sessions for KS (blue/dark blue) and IW (red/dark red), with SD error bars across sessions. KS was faster than her controls in all conditions (black outline and star), IW only differed in 2/8 valid trial conditions (black outlines and stars)

To simplify these complex comparisons, we repeated the analysis considering only valid-cue trials (blue and red bars, Fig. 11). Again, there was a significant difference between the two control groups [*F*(1,13) = 12.56, *p* = 0.004] with considerably shorter RTs for the younger controls (440 ms, SEM 21 ms compared to 542 ms, SEM 20 ms). For both control groups, the modulation of RT with location relative to the hand was stronger for the visible hand condition (compare left and right halves of Fig. 11).

Critically, there was also the expected interaction of vision, location, and laterality [*F*(1,13) = 10.11, *p* = 0.007] with shorter RTs in trials with ipsilateral compared to contralateral presentation of the target, in peri-personal space with vision (left-most columns, Fig. 11). Surprisingly, these effects reversed in extra-personal space, such that RTs were also short for the contralateral targets in extra-personal space. In addition, the pattern seen across the four vision conditions was weaker and reversed in the no-vision conditions, such that RTs were longer in ipsilateral peri-personal space than in contralateral peri-personal space (dark colours, Fig. 11). All other contrasts of the ANOVA were not significant (*p* > 0.13). However, this interaction also indicates that RTs were low for the contrasting condition, when the target was contralateral and in extra-personal space, with vision.

When we considered only invalid trials for the controls (not shown), only the age group factor was significant [*F*(1,13) = 7.79, *p* = 0.015] reflecting the slower RTs of the older group (565 ms, SEM 21 ms, compared to 479 ms, SEM 23 ms).

For the deafferented participants, we were able to test their performance over 3 (KS) or 4 (IW) sessions. Comparing their mean RTs against the separate control groups in two-way case–control comparisons, KS was significantly faster than the young control group in all 16 conditions, (*Q*’(6) < − 2.25, *p* < 0.012), while IW’s RTs were close to those of the older control group in all conditions (with only 4/16 comparisons significant, |*Q*’(6)|> 1.94, *p* < 0.03).

The case–control *Q*’ test allows us to test if the pattern of change across conditions is equivalent. KS showed only a main ‘group’ effect of shorter RTs compared to her controls (KS 360 ms, SEM 18 ms; controls 479 ms, SEM 23 ms; *Q*’(1) = 5.71, *p* = 0.017), and no differences across vision or cue validity factors compared to the controls (*Q*’(6) < 1.58, *p* > 0.9). However, IW, in his case–control comparison, showed difference from his controls in only 4 of the 16 individual conditions, and neither the main ‘group’ factor (IW 546 ms, SEM 15 ms; controls 565 ms, SEM 21 ms) nor the experimental factor differences was significant (*Q*’ < 2.38, *p* > 0.19); in other words, IW differed from his controls in showing very limited modulation of reaction times across conditions, whereas KS showed similar modulation to controls, but was overall faster.

**Fig. 12:**
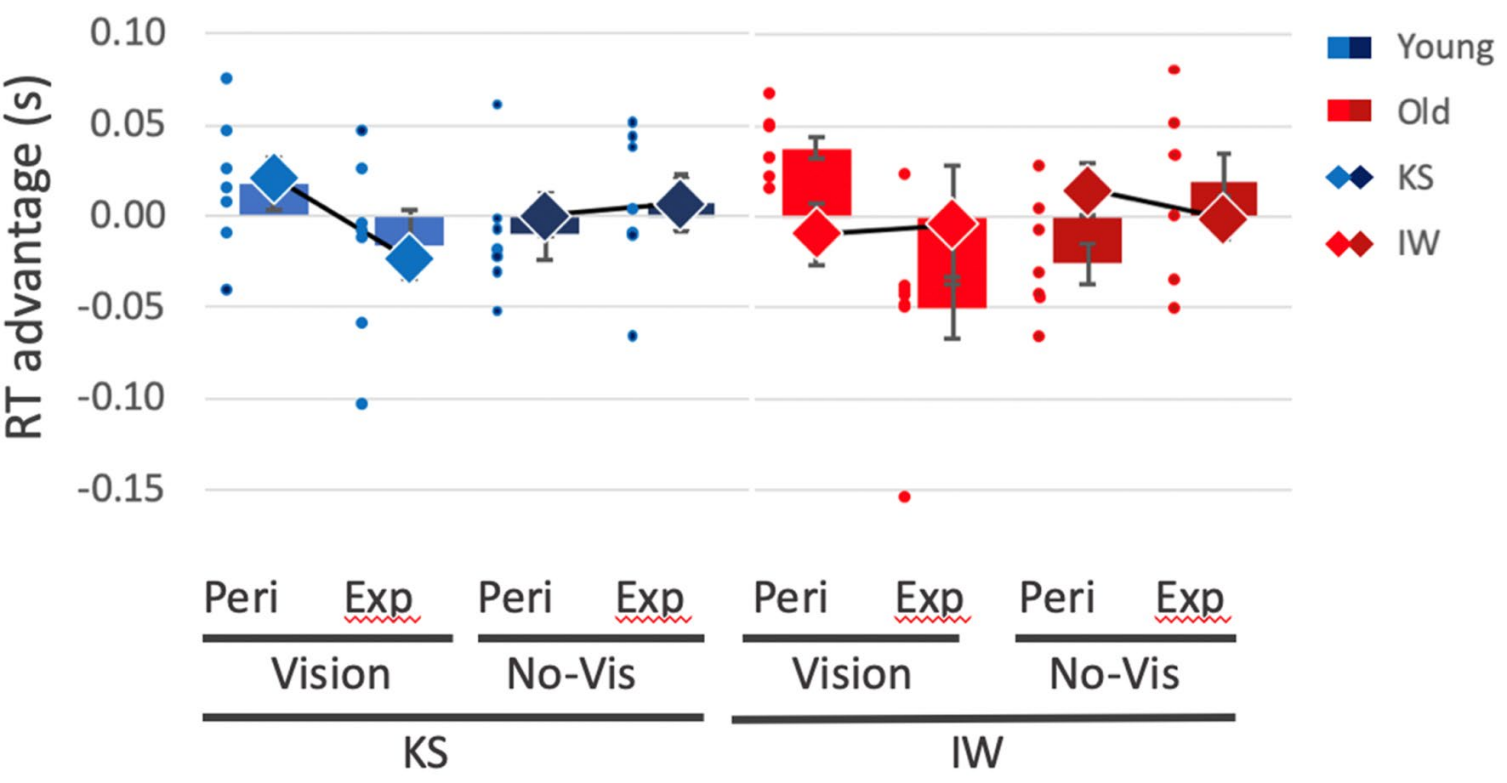
Reaction time differences between contralateral and ipsilateral target presentations, for valid trials only. Positive values represent an RT advantage for targets closer to the hand; negative values represent an advantage for targets contralateral to the hand. Blue and red bars are the group means (with ± 1 SEM) for the younger and older controls, respectively; small dots are the individuals’ data (*n* = 7 per group). Large diamonds are the means for KS and IW, averaged across session, ± 1 SD. Controls (and KS) show a reversal of RT advantage between peri-personal space (Peri) and extra-personal space (Exp) in vision (light blue/red) vs no-vision conditions (dark blue/red); IW showed no differences

The effects of the experimental factors (vision, target location, and laterality) were then analysed by running separate ANOVAs for KS and IW, across the 3 or 4 sessions. KS showed a significant vision–location–laterality interaction [*F*(1,2) = 25.1, *p* = 0.038] similar to that seen in the controls. This was particularly obvious in trials with the hand visible (light blue diamonds, Fig. 12). No other effects or interactions were significant for her (*p* > 0.15). IW did not show any significant differences across the factors (ANOVA, *p* > 0.137), thus differing from controls and from KS.

In summary, for both control groups and for KS, there were reaction time advantages for trials with targets ipsilateral to the visible hand in peri-personal space and disadvantages for targets in extra-personal, contralateral space (Fig. 12). These effects were weaker and the trends reversed for trials with the hand hidden beneath the occluding mirror, for both control groups and for KS. IW did not show significant RT differences across any conditions.

## General discussion

In this paper, we report four experiments that explore the maintenance of a body representation in two individuals living with chronic or congenital absence of somatosensation. We used perceptual tasks to probe judgements about hand shape, (Experiments 1A and 1B), arm reach (Experiment 2), and to test for an attentional bias in peri-personal space (Experiment 3).

Experiments 1 and 2 required conscious judgements about body size and shape, or the location of landmarks on the hand. We found strikingly different degrees of distortion between controls, and the participants, suggesting that the visual representation of hand shape may be quite distinct from metrical knowledge (Longo and Haggard 2010, 2012). Both IW, who has lived nearly 50 years without large fibre somatosensation from age 19, and KS, who was born and has lived 40 years without both large and small fibre somatic input from her body, performed these tasks well. While there were differences in their estimates across conditions, which we will discuss below, they were able to make consistent decisions in these tasks, and hence do have a conscious representation of their unfelt bodies. Vision seems to be sufficient for them to make reliable judgements in these circumstances. The absence of small diameter fibres in KS is not expected to be relevant here as these are not thought to contribute greatly to the body image or schema (Proske and Gandevia 2012). Thus, our study of two rare individuals demonstrates that the conscious body image can be developed and maintained even when without somatic sensation.

### Hand shape is accurately recognized with vision

In Experiment 1A, participants selected among a set of images of their own hand, distorted in length or width, the one closest to their perception of its real shape. The majority of control participants showed a systematic bias, choosing an image with reduced length/width ratio, on average 5% shorter and 5% wider, than the actual. While the results from the two test participants exposed differences between their two hands, in all cases, IW and KS showed less of a shortening than did controls, with KS even showing an overestimate of the length of her non-dominant hand. Thus, they both make “depictive” assessments (Longo and Haggard 2012) of the shape of their unseen hands that are—for the non-dominant hand—significantly more accurate than the controls. Whether this task assesses own-body image is, however, debatable and, in retrospect, it would have been useful to test participant’s sensitivity to distortion of others’ hands versus their own. If the identity of the hand is not critical, it may be that the task instead probes a form of mental imagery (Sirigu and Duhamel 2001; ter Horst et al. 2012).

### Reporting landmark positions on the hand reflects experience

In Experiment 1B, participants reported the location of landmarks on their unseen hands. The controls showed the previously described severe underestimation of finger lengths (Longo and Haggard 2010, 2012). We did not, however, reproduce the graduated and increasing underestimation from thumb to little finger that Longo and colleagues report, possibly due to a difference in instructions. Longo and colleagues instructed their participants to report the felt location of the hand landmarks; since KS and IW would not be able to feel their hands, our instruction was to place the cursor onto the hand landmark, and we did not mention the felt position.

Interestingly, Ganea and Longo (2017) showed that the maps were robust to whether the hand was directly under the report surface or held on the lap. Our tests with IW and KS confirm that the reported positions are similar regardless of actual hand location. This suggests that the task is heavily reliant on a visual (KS and IW) or visuo-somatic representation (controls). Longo and Haggard (2010, 2012) have suggested that the “metrical” graduated distortion might be related to the different density of cutaneous receptors across the hand. Since the conscious representation of body shape is normally multi-modal, integrating visual and somatosensory inputs, the contribution of these two sources might alter across the cortical hand representation, affecting overestimation in their judgements. Instead, we found that in both control groups, all-finger digits were estimated at between 35 and 40% of their actual length (although the thumb was less distorted, at about 45%), while hand width was also slightly underestimated at about 90% of true width. The overall length/width ratio is consistent with—but much more marked than—the average selected ratios of less than unity in Experiment 1A. Thus, granular support for the influence of receptor density on hand representation was not found.

Cocchini et al. (2018) have reported that hand maps reflect dextrous hand use and are more accurate in trained magicians. The reduced error for thumb length that we observed is consistent with this idea, given the thumb’s importance in everyday actions and its priority in placement for grasp and manipulation of objects (Smeets and Brenner 1999; Smeets et al. 2002). The dominance of the thumb is further evident in both motor and sensory homunculi (Penfield and Boldrey 1937). There is also typically less individuated use of the lateral fingers (Miall et al. 2019), and in a grasping action, these can be guided by somatic rather than visual control—potentially leading to greater distortion in our visually based mapping experiment.

IW and KS differed somewhat from controls in their distortion patterns. They underestimated digit lengths, though by less than the controls, meaning that they had greater accuracy, while slightly overestimating hand width. These metric distortions were evident and consistent for both hands, with a suggestion of greater accuracy for the non-dominant hand. We had expected that due to their greater reliance on vision for control of action and their lack of access to a topographically skewed somatosensory input, IW and KS might be more veridical in their reports of the individual digit lengths than controls. This was indeed the case for IW. On the other hand, the shortening of digit lengths that KS displayed was similar to that seen in the controls, calling into question a simple somatosensory representational argument for the under-representation of digit length.

The reporting procedure used by KS and IW, verbally instructing the experimenter to steer the on-screen cursor, was clearly distinct from the self-driven joystick movement that the controls used. Note, however, that the joystick provided velocity control over the cursor position, so the controls gained no direct proprioceptive feedback of selected position. In addition, Longo (2018) compared the maps generated by participants using either a long pointer to indicate the landmarks, or verbal instruction of the experimenter who held the pointer; the maps were similar, and the pattern of distortion equivalent. Hence, we do not think the mode of reporting explains differences between IW, KS, and the controls.

The slight underestimates of hand width (between the primary knuckles for the index and little fingers) seen in our control groups are in contrast to the overestimation reported by Longo and colleagues (Longo and Haggard 2010, 2012; Ganea and Longo 2017). They asked participants to use (or to guide) a long-thin pointer to mark a position on a surface a few centimetres above the hand, and to move it to the lateral edge of the board between trials. We used a cursor that always originated at the centre of the lower edge of the screen, and the screen image was coplanar with the table top on which the hand rested. However, Longo and Haggard (2010) have shown that hand maps are unaffected if the hand is rotated 90 degrees, ruling out perspective biases. It may be therefore that a key difference is in guiding a visual cursor to each landmark, rather than using a pointer.

A surprise was that KS was initially uncertain about the names of her fingers. This suggests that she has paid little attention to her hands. KS does not use her hands much and rarely uses the lateral digits (middle, ring, and little). There are clear abnormalities in the musculoskeletal arrangement of her hands, including an inability to fully extend at the wrist, and in their central control, since she cannot independently move the middle, ring, or little fingers on either hand. There are also clear differences between IW and KS in their activities of daily living (see ABILHAND scores, Methods). She does not perform most dextrous tasks such as cutting up food, buttoning, brushing teeth, and combing her hair. In tasks such as using a spoon (which KS does daily) or writing (which she does rarely) KS uses an all-finger power grasp. In tasks such as picking up cards or a jigsaw puzzle piece, she uses a precision grip between thumb and index finger.

In contrast to KS, IW has used his hands extensively since an intense period of re-learning and rehabilitation just months after the onset of his neuropathy (Cole 1995). He daily performs tasks such as cooking, dressing, and so on. He is competent in many grasp actions, albeit with modifications in hand posture to improve object stability: he mainly uses his thumb, index, and middle fingers and often actively excludes his lateral fingers from the grasp, either extending them or flexing them into the palm of his hand (Miall et al. 2019). It is striking that the digit length estimates derived from his hand maps were more accurate than KSs and the controls (Fig. 4). He also had a more accurate spatial representation of his hands (Figs. 6 vs 5), for example with the angles between the knuckles closer to reality than for most controls. Hence, it is possible that chronic absence of somatosensory inputs together with visually controlled hand movement led to an accurate, visually based representation in IW, compared to controls whose body image is distorted by somatosensory inputs. Supporting this, Longo et al. (2012) reported that a subject born without one arm estimated the digit lengths more accurately for her phantom than her intact hand, consistent with a lack of distorting somatosensation from the missing limb facilitating veridical body image. Knowledge of hand shape gained from daily use of the contralateral, intact hand may have further augmented a veridical image. Interestingly, IW is strongly left-hand dominant, and yet this did not lead to perceptual differences between hands.

One explanation for the limited difference in KS’s hand maps from controls is the opposing effects of her lack of somatic input, taking her closer to veridical (as in IW) and her lack of motoric use, potentially augmenting the distance from veridical. Hence, we suggest that it may be KS’s impoverished hand use that has led to her degraded metrical knowledge of shape and size of her hands compared to IW. Her foreshortened hand map reflects her lack of dextrous experience tempered by a lack of somatosensory inputs, whereas the even greater foreshortening in controls is due to the presence of somatosensory distortions.

### Overestimation of arm length

Healthy controls typically overestimate the target distance they can reach (Carello et al. 1989; Bootsma et al. 1992; Heft 1993; Rochat and Wraga 1997; Mark et al. 1997; Leclere et al. 2019). In the judgement of arm length, estimated in Experiment 2, we found that this overestimation was even greater for IW than for his controls, whereas the overestimation for KS was comparable to that of younger controls. The high variability of the original control group for KS, and the low mean bias of the older control group, was highlighted when testing a large group of young undergraduates. In that comparison, both IW’s and KS’s reach estimation biases were similar to the mean of both the undergraduate and older control groups (Fig. 9a).

Both KS and IW reported thinking through and attempting to use surrounding landmarks in making these judgements. IW’s precision in his reaching judgements, based on the slope of the psychometric curve, was slightly lower than that of the controls, i.e., the JND was larger, even more so for his non-dominant arm. This might again reflect a motoric component; his visual calibration of reach distance is better for his more used arm. KS’s JND values were not significantly different from the controls, however. When we repeated the measurements after adding a wider surround to the reaching arena, so that any obvious landmarks were distant from the visual target, their accuracy was unchanged, albeit this was only tested after they had familiarisation with the paradigm. It is also interesting to note that despite her self-reported poor depth perception, KS performed as well as controls. It remains to be seen how extra-personal depth perception in KS and IW compares to controls.

### Attentional bias to peri-personal space

In Experiment 3, testing reaction times to detect visual targets, we found the expected RT advantages in our control participants for targets ipsilateral to the visible hand in peri-personal space relative to contralateral targets. This is consistent with the previously reported attentional advantage for targets close to the hand, within peri-personal space (Reed et al. 2006; Brown et al. 2015), that is thought to depend on multi-modal integration of visual, haptic, and proprioceptive representations in parietal cortex. Unexpectedly, and unlike previous reports (Reed et al. 2006; Brown et al. 2015), we found this effect reversed in extra-personal space, beyond reaching distance, for both control groups. It also reversed when the hand was hidden, although the depth of attentional modulation was less (Fig. 11, right side). We cannot yet explain these reversals, which are not expected in an account based simply on hand–target proximity. However, others have suggested that competitive attentional processes are at play. Hand–target proximity reduces reaction times in a visual search paradigm, but it simultaneously increases the difficulty of disengagement and relocation of attention from one place to another (Thomas and Sunny 2017).

Regardless of the attentional mechanism, we can safely assume that differences in RT depend on the relative distance between the target and the hand (which was not moved in our paradigm), as they were found for the controls when the hand was both visible and hidden. Thus, we can use the task to probe whether IW and KS also display these differences. KS showed significant RT difference in the visual but not non-visual conditions; IW showed no significant differences in either condition. These data suggest that KS has a visually based body representation or schema, whereas IW has no discernible body-schema-based representation of peri-personal space.

Two features of KS’s data are striking. First, her reaction times were uniformly short, when compared to the young controls, and second, as mentioned above, in the hand-visible conditions she showed the same pattern of modulated RTs as the controls. Fast responses in these attentional detection tasks are thought to reflect implicit and bottom–up processes (Risko and Stolz 2010). She may have used a predominantly bottom-up process because, unlike the other tasks, the visual detection task did not require mental imagery of her body. Additionally, a simple and consistent verbal response was sufficient, rendering her performance unaffected by her dextrous inexperience. That her RTs were modulated by target–hand distance suggests this bottom–up approach is influenced within peri-personal space, presumably by enhanced visual representation (Brown et al. 2015). This interpretation is further supported by the finding that RT modulation was only present when KS’s hand was visible: when her hand was occluded, RTs were unmodulated. These data argue that KS has a vision-based subconscious representation of space around her body.

In contrast to KS, IW was slow in his reaction times, even compared to the older control group. Additionally, he did not show any significant modulation of RTs across the tested conditions (other than the typical slow responses for invalid cue conditions, which we do not report here). We suggest that he was more strategic, top–down, in his approach—and possibly more cautious about the invalid and catch trials—as he was in a previous attentional task (Nougier et al. 1994). IW prefers a cautious approach in tasks that he wishes to perform as well as possible (Renault et al. 2018). There were certainly no signs of any subconscious proximity advantage for IW.

### Visual proprioception versus visual control

Another way of framing the distinction between IW and KS is that KS has a strong sense of “visual proprioception” (Lee and Lishman 1975), the unconscious visual representation of the body, whereas IW does not. Again, while speculative, it is possible that visual inputs have replaced somatic inputs in KS’s central representations at some point in her development, and she may be able to use such alternative pathways (cerebellar and/or cerebral) without need for cognitive attention. In contrast, such a replacement would not have occurred in IW who matured into adulthood with intact somatosensation. Instead, IW appears to have replaced his loss of somatic input with conscious strategic control. Revised versions of Experiments 1 and 2 in which judgements of body shape and size are made implicit might be able to determine whether KS, unlike IW, may also have developed the capacity for visually controlled non-conscious motoric judgements. Other recent experiments support the idea that KS has more automaticity and less conscious visual control of hand movements than IW (Miall et al., in revision).

While visual proprioception may fuel KS’s automaticity and rapid responses, it is not sufficient to produce representational accuracy, which may in turn be reduced by her limited motor experience. In contrast, IW’s awareness of his body is entirely top–down, constructed through comparatively slow information processing traversing (we propose) conscious visual streams. IW is clear about his need to be consciously aware of his body position to control movement, and its dependence on conscious vision: “everything is through vision” (Cole 2016).

Finally, we note that our assessments of hand configuration and arm reach (Experiments 1 and 2) provide objective evidence that both IW and KS have developed and maintained a perceptually accessible representation of the body, or a body image. As IW relates, “rather than being disembodied, I am completely, totally, embodied. If I was not I would not know where I am. I re-associate and reconnect constantly.” (Cole 2016). KS has been asked repeatedly about her sense of the body. She never hesitates and has always maintained that she has one; when she closes her eyes, the world goes away, but she does not. To illustrate this difference, upon awakening and opening their eyes in the morning, IW goes through a process of re-establishing where his body is, whereas KS simply welcomes back the world to her embodied self.

In sum, and returning to the conceptual framing provided by body image and body schema, our data lend support to the idea that KS has developed a low-fidelity, automated (fast) motor representation (or schema) whereas IW uses a slow, high-fidelity, cognition-dependent representation for movement control.

## Data Availability

The datasets generated or analysed during the current study are available from the corresponding author on reasonable request.
